# Ludangshen oral liquid for treatment of convalescent COVID-19 patients: a randomized, double-blind, placebo-controlled multicenter trial

**DOI:** 10.1186/s13020-022-00602-x

**Published:** 2022-04-02

**Authors:** Xuedong An, Bo Peng, Xiaodong Huang, Hongmei Jiang, Zhang’e Xiong, Hong Zhang, Fengmei Lian, Yuanming Ba, Xiaolin Tong

**Affiliations:** 1 Guang’an Men Hospital of China Academy of Chinese Medical Sciences, Beijing, 100053 China; 2 Wuhan Third Hospital, Wuhan, 430060 China; 3 Hubei Provincial Hospital of Traditional Chinese Medicine, Wuhan, 430061 China

**Keywords:** Convalescent COVID-19, Ludangshen oral liquid, Traditional Chinese medicine, Digestive function, Randomized controlled trial

## Abstract

**Objective:**

To explore the effect of Ludangshen oral liquid for treatment of convalescent patients with coronavirus disease 2019 (COVID-19) with randomized, double-blind, placebo-controlled multicenter method.

**Methods:**

200 convalescent COVID-19 patients who had symptoms related to decreased digestive and respiratory function were randomly divided to either receive Ludangshen oral liquid or placebo for 2 weeks. The severity of clinical symptoms including fatigue, anorexia, abdominal distension, loose stools, and shortness of breath were assessed by visual analogue scale and observed at before and after treatment. The improvement and resolution rates of clinical symptoms were evaluated. Full analysis set (FAS) and per-protocol set (PPS) were used for statistical analyses. Adverse events were recorded during the study.

**Results:**

8 patients did not complete the study. After 2 weeks of treatment, both FAS and PPS results showed that patients in Ludangshen group had significantly lower score of fatigue, anorexia, loose stools, and shortness of breath than placebo group (*P* < 0.05), while there was no significant difference in distention (*P* > 0.05). The improvement rate of fatigue, anorexia, distension, loose stools and shortness of breath were significantly higher in Ludangshen group (*P* < 0.05), as well as the resolution rates (*P* < 0.05) except for shortness of breath (*P* > 0.05). There were two cases of adverse events, with one nose bleeding in Ludangshen group and one headache in placebo group.

**Conclusion:**

The study suggested that two weeks of Ludangshen oral liquid treatment may have certain effects for convalescent COVID-19 patients on improving digestive and respiratory symptoms including fatigue, anorexia, loose stools and shortness of breath, which may be one of the choices for management of convalescent COVID-19 patients with digestive and respiratory symptoms.

## Introduction

The coronavirus disease 2019 (COVID-19) pandemic is still severe all around the world, with over 472 million cases and 6 million deaths cumulatively reported by World Health Organization till March 24, 2022 [1]. In China, traditional Chinese medicine (TCM) together with Western medicine has been highly recommended in prevention, and treatment of COVID-19 [2, 3]. TCM has played an important role in relieving symptoms of COVID-19 patients as well as delaying or reducing the progression from mild to severe condition [4]. As a result, the epidemic has almost been controlled in China [5].

Till January 17, 2022, almost 123,169 COVID-19 patients have been cured and discharged from hospitals in China [6]. For convalescent patients, who had met the discharge criteria (absence of clinical symptoms and radiological abnormalities, as well as two consecutive negative test results of real time reverse transcriptase-polymerase chain reaction (RT-PCR)) [2], about 10% patients may still experience mild clinical discomforts such as cough and fatigue, especially those who were severe or critical cases [4, 7, 8]. As the number of patients who are discharged from hospitals or discontinued of quarantine has increased rapidly, the management of convalescent COVID-19 patients has gradually become a new urgency [4]. In TCM theory, COVID-19 belongs to “cold-damp pestilence” category [9]. The pathogenic cold-damp together with epidemic pathogen firstly invaded the exterior and block the lung*-wei*; as the lung meridian originate from middle-*jiao*, the function of spleen and stomach were then affected [4, 10]. Therefore, the patients during recovery period would mainly present clinical symptoms related to decreased digestive and respiratory function, such as fatigue, anorexia, diarrhea, loose stools, and shortness of breath, which should be highly managed [4, 10]. TCM has been highly recognized for their remarkable effects in fighting infectious diseases. Acted as "preventive treatment of diseases", it has advantages in preventing disease recurrence after recovery [11]. TCM can provide comprehensive rehabilitation therapies including herbal medicine as well as non-pharmacological therapies (acupuncture, taichi) to improve clinical symptoms of COVID-19 patients during recovery period. In this study, we investigated the effect of Ludangshen oral liquid, a Chinese patent medicine, for treatment of digestive and respiratory symptoms in convalescent COVID-19 patients, in order to provide clinical evidence for the treatment of COVID-19.

## Method

### Study design

The study was designed as a multi-center randomized, placebo-controlled study in patients during recovery period of COVID-19 treated with Ludangshen oral liquid. The trial was registered with the China Clinical Trial Registration Center (No. ChiCTR200003291). The trial was approved by the Ethics Committee (No. KY2020-050). The written informed consent was signed by all patients.

The diseases involved in this study have no previous relevant research data for reference, and none of the intervention measures have reference efficacy and effect values. Combined with the opinions of statistical experts, the subjects were randomly divided into the treatment group and the control group according to 1:1, and the sample size of each group is estimated to be 100 cases, with total of 200 cases.

### Patients

The convalescent COVID-19 patients with lung-spleen qi deficiency syndrome in presence of digestive and respiratory symptoms, were enrolled from Hubei Provincial Hospital of Traditional Chinese Medicine, Ezhou Traditional Chinese Medicine Hospital, and Xiaogan Traditional Chinese Medicine Hospital.

The convalescent patients were defined as those who have met the criteria of medical isolation and discharge [3]. The criteria was: (1) body temperature returned to normal for more than three days; (2) respiratory symptoms improved significantly; (3) lung imaging showed obvious absorption; (4) two consecutive negative test results of nucleic acid (the sampling interval was at least one day) [2].

The inclusion criteria were as follows: (1) meeting the criteria of medical isolation and discharge mentioned above; (2) meeting the criteria of lung-spleen qi deficiency syndrome (Clinical manifestations: shortness of breath, fatigue, anorexia, nausea, fullness, loose stool, and uneasiness. The tongue is pale and greasy.); (3) at least two or three TCM symptoms including fatigue, anorexia, diarrhea, loose stools, shortness of breath, or a single symptom score greater than 4 points when measured by a visual analogue scale of 0–10 points; (4) discharged from hospital for two to four weeks; (5) aged 18 to 70 years; (6) provision of written informed consent.

The exclusion criteria were as follows: (1) presence of symptoms related to decreased digestive function caused by other reasons or other digestive system diseases; (2) having difficulty in oral medication due to underlying diseases or other reasons; (3) having severe basic diseases, such as uncontrolled heart, lung, kidney, digestive diseases, hematological diseases, neuropsychiatric diseases, immune diseases, metabolic diseases, malignant tumors, and severe malnutrition; (4) allergy to the examined treatments, or with allergic constitution; (5) women in pregnancy or lactation; (6) having mental diseases; (7) participating in other clinical trials.

### Treatment

Patients in intervention group were treated with Ludangshen oral liquid (Shanxi Zhenglai Pharmaceutical Co., Ltd., Shanxi, China, production batch No. 2003305), 10 ml twice daily for 2 weeks. The composition of Ludangshen oral liquid is ludangshen. Patients in control group were treated with placebo of Ludangshen oral liquid (Shanxi Zhenglai Pharmaceutical Co., Ltd., Shanxi, China), 10 ml twice daily for 2 weeks. The Ludangshen oral liquid and placebo solution were indistinguishable regarding flavor, taste, and appearance including color and packaging. Patients who have basic diseases were allowed to take corresponding treatments.

### Outcomes

The changes in digestive and respiratory symptoms, including fatigue, anorexia, abdominal distension, loose stools and shortness of breath, were observed. The visual analogue scale (VAS) of 0 to 10 score was used to evaluate the severity of symptoms; “0” represents asymptomatic symptoms, while “10” represents the most severe symptoms. The higher the score, the more severe the symptoms. Based on the VAS score, the improvement and resolution rates of symptoms were assessed. Adverse events were recorded during the study period.

### Randomization and blinding

Eligible patients were randomized (1:1) to receive either Ludangshen oral liquid or placebo. The randomization was performed using the clinical research central randomization system (online version). The doctors, patients, outcome investigators and statistics were blinded to the treatment assignments of patients, only if emergency happened.

### Statistical analysis

Data were analyzed by SPSS 26.0 software. Continuous variables were presented as mean ± standard deviation (SD) if normally distributed, or as median with interquartile range (IQR) if not normally distributed. Categorical variables were presented as the number and percentage (%). The t-test was used to compare continuous data between groups if data were normally distributed, otherwise two independent sample rank sum test (Mann–Whitney U test) was used. The X^2^ test or Fisher’s exact test was used to compare categorical data between groups. All statistical tests were two-sided, and a P value less than or equal to 0.05 was considered as statistically significant.

Two analysis sets including full analysis set (FAS) and per protocol set (PPS) were analyzed for effectiveness in this study. FAS was used to analyze the data of patients who went through randomization and received treatments; for missing data, last time measurement was carried forward and filled. PPS was used to analyze the data of patients who fully completed the study with good compliance.

## Results

### Baseline characteristics

**Fig. 1:**
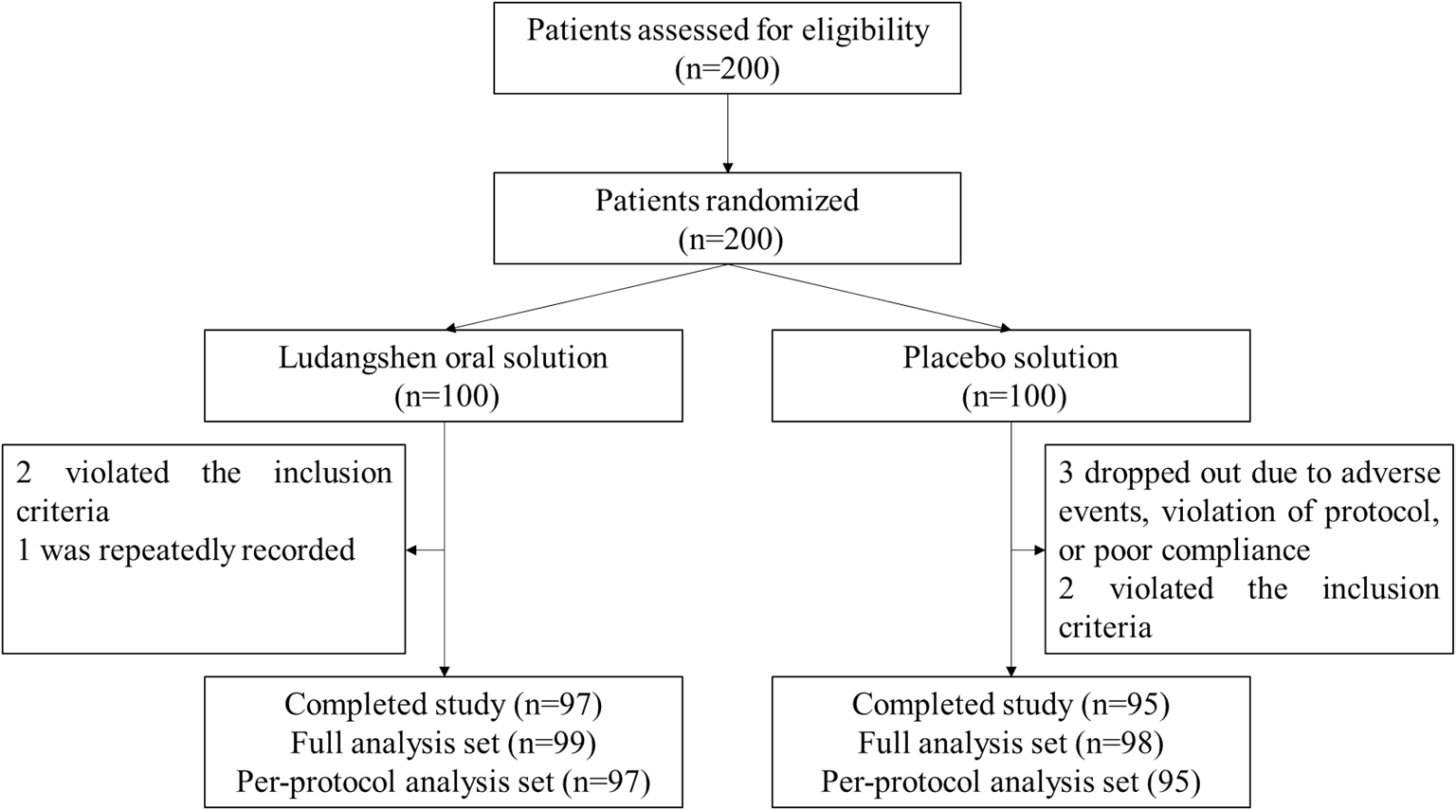
Enrollment of the patients and completion of the study

A total of 200 convalescent COVID-19 patients from three centers were enrolled and assessed for eligibility, with 100 patients in each group after randomization. Three patients dropped out of the study because of poor compliance, violation of the treatment protocol, or adverse events; four patients did not meet the inclusion criteria, one patient repeatedly recorded. Totally, 192 patients fully completed the study (Fig. 1).

The demographic and baseline characteristics were shown in Table 1. There was no significant difference in gender, age, smoking, drinking, drug allergy history, systolic blood pressure, diastolic blood pressure, resting heart rate, breathing, CT imaging, hospitalization length, discharge length, comorbidities and concomitant medication between groups (*P* > 0.05). The body mass index value was significantly lower in Ludangshen group than control group (*P* = 0.021).

**Table 1:** Demographic and baseline characteristics of patients between Ludangshen and placebo group

Characteristics	Ludangshen (n = 99)	Placebo (n = 98)	t/χ2/Z	*P* value
Male (n, %)	28 (28.3)	33 (33.7)	0.747	0.387
Age (n, %)			− 0.191	0.848
≤ 30	9 (9.1)	5 (5.1)		
31–40	16 (16.2)	23 (23.5)		
41–50	23 (23.2)	21 (21.4)		
51–60	25 (25.3)	18 (18.4)		
≥ 61	26 (26.3)	31 (31.6)		
BMI (Mean ± SD)	22.7 ± 2.86	23.7 ± 2.97	− 2.236	**0.021**
Smoking (n, %)	5 (5.0)	9 (9.2)	1.138	0.251
Drinking (n, %)	4 (4.0)	9 (9.2)	2.168	0.141
Temperature (Median, IQR)	36.5 (36.3–36.5)	36.5 (36.3–36.5)	− 0.048	0.962
SBP (Mean ± SD)	123.4 ± 11.2	124.2 ± 11.7	− 0.512	0.609
DBP (Median, IQR)	79 (73–81)	76 (70–80.25)	− 0.799	0.424
Breathing (Median, IQR)	18 (18–19)	18 (18–19)	− 0.434	0.664
Resting heart rate (Median, IQR)	80 (75–86)	79.5 (75–86)	− 0.498	0.625
Clinical classification (n, %)			− 0.296	0.767
Mild cases	10 (10.1)	7 (7.1)		
Moderate cases	77 (77.8)	80 (81.6)		
Severe cases	11 (11.1)	11 (11.2)		
Critical cases	1 (1.0)	0 (0.0)		
CT imaging (n, %)			0.359	0.549
Normal	19 (19.2)	22 (22.5)		
Abnormal	80 (80.8)	76 (77.6)		
Blood routine (n, %)			1.221	0.269
Normal	99 (100.0)	94 (95.9)		
Abnormal	0 (0.0)	1 (1.0)		
Allergy history (n, %)	16 (16.3)	14 (14.3)	0.133	0.737
Comorbidities (n, %)	25 (25.3)	27 (27.6)	0.166	0.683
Initial symptoms (n, %)				
Fatigue	94 (94.9)	90 (91.8)	–	–
Anorexia	68 (68.7)	70 (71.4)	–	–
Distention	43 (43.4)	34 (34.7)	–	–
Loose stools	48 (48.5)	52 (53.1)	–	–
Shortness of breath	50 (50.5)	50 (51.0)	–	–
Concomitant medication (n, %)	13 (13.1)	15 (15.3)	0.217	0.641
Hospitalization length (Median, IQR)	19 (11–28)	18 (10.75–26.5)	− 0.23	0.818
Discharge length (Median, IQR)	71 (59–81)	69.5 (50–80)	− 0.61	0.542

*BMI* body mass index, *SD* standard derivation, *IQR* interquartile range, *SBP* systolic blood pressure, *DBP* diastolic blood pressure

### Comparison of symptom scores

At week 1, fatigue score was significantly lower in Ludangshen group compared to that in placebo group (FAS: *P* < 0.001; PPS: *P* < 0.001). Ludangshen group had significantly lower symptom score in anorexia, distension, loose stools and shortness of breath compared to placebo group for FAS analysis, but the PPS analysis showed not significantly difference between groups (FAS: *P* < 0.001, *P* = 0.024, *P* = 0.001, *P* = 0.016; PPS: *P* = 0.535, *P* = 0.619, *P* = 0.749, *P* = 0.996) (Table 2).

**Table 2:** Comparison of symptom scores between Ludangshen and placebo group

Variables	FAS analysis			PPS analysis		
	Ludangshen	Placebo	*P* value	Ludangshen	Placebo	*P* value
Fatigue (n)	94	90		92	92	
Week 1	2.88 ± 1.44	3.64 ± 1.43	** < 0.001**	2.87 ± 1.45	3.51 ± 1.55	** < 0.001**
Week 2	1.31 ± 1.42	3.21 ± 1.85	** < 0.001**	1.35 ± 1.46	3.11 ± 1.92	** < 0.001**
Anorexia (n)	68	70		67	68	
Week 1	1.66 ± 1.17	2.61 ± 1. 53	** < 0.001**	1.66 ± 1.74	2.62 ± 1.55	0.535
Week 2	0.78 ± 1.22	2.09 ± 1.74	** < 0.001**	0.78 ± 1.23	2.09 ± 1.76	** < 0.001**
Distention (n)	43	34		42	43	
Week 1	2.02 ± 1.88	2.32 ± 2.10	**0.024**	2.00 ± 1. 90	2.27 ± 2.11	0.619
Week 2	0.74 ± 1.62	1.53 ± 1.89	0.987	0.74 ± 1.64	1.58 ± 1.90	0.341
Loose stools (n)	48	52		48	50	
Week 1	2.02 ± 1.77	2.65 ± 1.94	**0.001**	2.02 ± 1.77	2.70 ± 1.96	0.749
Week 2	1.04 ± 1.91	2.12 ± 2.37	**0.001**	1.04 ± 1.91	2.20 ± 2.37	**0.001**
Shortness of breath (n)	50	50		49	49	
Week 1	3.40 ± 1.36	4.02 ± 1.65	**0.016**	3.43 ± 1.35	4.02 ± 1.66	0.996
Week 2	1.76 ± 1.38	3.26 ± 2.07	**< 0.001**	1.78 ± 1.396	3.27 ± 2.09	** < 0.001**

*FAS* full analysis set, *PPS* per protocol set

At week 2, patients in Ludangshen group had significantly lower score of fatigue, anorexia, loose stools, and shortness of breath (FAS: *P* < 0.001, *P* < 0.001*, P* = 0.001, *P* < 0.001; PPS: *P* < 0.001, *P* < 0.001, *P* = 0.001, *P* < 0.001). There was no significant difference in distension score between the two groups (FAS: *P* = 0.987; PPS: *P* = 0.341) (Table 2).

### Comparison of symptom improvement rate

At week 1, the improvement rates of fatigue and anorexia were significantly higher in Ludangshen group than placebo group (FAS: *P* = 0.002, *P* < 0.001; PPS: *P* = 0.003, *P* < 0.001). Patients in Ludangshen group also had obviously higher improvement rate in distension and loose stools by FAS analysis, but the differences were not significant by PPS analysis (FAS: *P* = 0.036, *P* = 0.039; PPS: *P* = 0.059, *P* = 0.062). For shortness of breath, there was no significant difference between groups (FAS: *P* = 0.23; PPS: *P* = 0.312). (Table 3).

**Table 3:** Comparison of clinical symptom improvement rate between Ludangshen and placebo group

Variables		FAS analysis			PPS analysis		
		Ludangshen	Placebo	*P* value	Ludangshen	Placebo	*P* value
Fatigue (n)		94	90		92	89	
Week 1		52 (55.3)	29 (32.2)	**0.002**	50 (54.3)	29 (32.6)	**0.003**
Week 2		81 (86.2)	38 (42.2)	** < 0.001**	78 (84.8)	37 (41.6)	** < 0.001**
Anorexia (n)		68	70		67	68	
Week 1		49 (72.1)	25 (35.7)	** < 0.001**	48 (71.6)	25 (36.8)	** < 0.001**
Week 2		61 (89.7)	38 (54.3)	** < 0.001**	60 (89.6)	36 (52.9)	** < 0.001**
Distension (n)		43	34		42	33	
Week 1		28 (65.1)	14 (41.2)	**0.036**	27 (64.3)	14 (42.4)	0.059
Week 2		38 (88.4)	22 (64.7)	**0.013**	38 (90.5)	21 (63.6)	**0.01**
Loose stools (n)		48	52		48	50	
Week 1		32 (66.7)	24 (46.2)	**0.039**	32 (66.7)	24 (48.0)	0.062
Week 2		41 (85.4)	32 (61.5)	**0.007**	41 (85.4)	30 (60.0)	**0.005**
Shortness of breath (n)		50	50		49	49	
Week 1		27 (54.0)	21 (42.0)	0.23	26 (53.1)	21 (42.9)	0.312
Week 2		44 (88.0)	28 (56.0)	** < 0.001**	43 (87.8)	28 (57.1)	**0.001**

*FAS* full analysis set, *PPS* per protocol set

At week 2, the improvement rate of fatigue, anorexia, distension, loose stools, and shortness of breath were all significantly higher in Ludangshen group compared to placebo group (FAS: *P* < 0.001, *P* = 0.013, *P* = 0.007, *P* < 0.001, *P* < 0.001; PPS: *P* < 0.001, *P* < 0.001, *P* = 0.01, *P* = 0.005, *P* = 0.001). (Table 3).

### Comparison of symptom disappearance rate

At week 1, the symptom disappearance rate of fatigue and anorexia were significantly higher in Ludangshen group than placebo group (FAS: *P* = 0.027, *P* = 0.036; PPS: *P* = 0.026, *P* < 0.001). For distension, loose stools, and shortness of breath, there were no statistically significant difference in symptom disappearance rate between groups (FAS: *P* = 0.194, *P* = 0.07, *P* = 1; PPS: *P* = 0.197, *P* = 0.058, *P* = 1) (Table 4).

**Table 4:** Comparison of clinical symptom disappearance rate between Ludangshen and placebo group

Variables	FAS analysis			PPS analysis		
	Ludangshen	Placebo	*P* value	Ludangshen	Placebo	*P* value
Fatigue (n)	94	90		92	89	
Week 1	5 (5.3)	0 (0.0)	**0.027**	5 (5.4)	0 (0.0)	**0.026**
Week 2	35 (5.3)	8 (8.9)	** < 0.001**	34 (36.7)	8 (9.0)	** < 0.001**
Anorexia (n)	68	70		67	68	
Week 1	10 (14.7)	3 (4.3)	**0.036**	10 (14.9)	3 (4.4)	**0.038**
Week 2	39 (57.4)	15 (21.4)	** < 0.001**	39 (58.2)	14 (20.6)	** < 0.001**
Distention (n)	43	34		42	33	
Week 1	10 (23.3)	4 (11.8)	0.194	10 (23.8)	4 (12.1)	0.197
Week 2	29 (67.4)	12 (35.3)	**0.005**	29 (69.1)	11 (33.3)	**0.002**
Loose stools (n)	48	52		48	50	
Week 1	11 (22.9)	5 (9.6)	0.07	11 (22.9)	5 (10.0)	0.058
Week 2	32 (66.7)	20 (38.5)	**0.005**	32 (66.7)	18 (36.0)	**0.002**
Shortness of breath (n)	50	50		49	49	
Week 1	0 (0.0)	0 (0.0)	1.000	0 (0.0)	0 (0.0)	1.000
Week 2	8 (16.0)	3 (6.0)	0.110	8 (16.3)	3 (6.1)	0.110

*FAS* full analysis set, *PPS* per protocol set

At week 2, the symptom disappearance rate of fatigue, anorexia, distension and loose stools were significantly higher in Ludangshen group than placebo group (FAS: *P* < 0.001, *P* < 0.001, *P* = 0.005, *P* = 0.005; PPS: *P* < 0.001, *P* < 0.001, *P* = 0.002, *P* = 0.002). There was no statistically significant difference in symptom disappearance rate of shortness of breath between groups (FAS: *P* = 0.11; PPS: *P* = 0.11) (Table 4).

### Safety evaluation

The adverse events were recorded during the study. There were two cases of adverse events. One case in Ludangshen group had nose bleeding, and one case in placebo group had headache. The rate of adverse events was 1% in both groups (*P* > 0.05). There were no significant differences in systolic blood pressure, diastolic blood pressure and resting heart rate at the end of treatment between Ludangshen and placebo group (*P* > 0.05).

## Discussion

In this study, Ludangshen oral liquid could significantly improve certain symptoms related to decreased digestive and respiratory functions. The FAS results showed that symptoms including fatigue, anorexia, loose stools and shortness of breath were improved after treatment of Ludangshen oral liquid compared to placebo at week 1 and week 2. The results were consistent with PPS analysis at week 2. However, for results at week 1, there was difference between FAS analysis and PPS analysis. In PPS analysis, only fatigue was shown to be significantly improved. It seems that week 2 result was more stable, and that compared to 1-week treatment of Ludangshen oral liquid, 2 weeks treatment indicated better effect. In terms of symptom improvement rate and is appearance rate at week 2, the results from FAS analysis and PPS analysis were consistent. Improvement rate of fatigue, anorexia, abdominal distension, loose stools and shortness of breath in COVID-19 patients were all significantly increased after treated by Ludangshen oral liquid. In addition, symptom disappearance rate of fatigue, anorexia, abdominal distention and loose stools were also significantly increased.

The main component of Ludangshen oral liquid is Dangshen (*Radix Codonopsis*), which is the dried root of Campanulaceae plants. As recorded in *Pharmacopoeia of people's Republic of China,* Dangshen is sweet and neutral, and can supplement the center to boost qi, and fortify the spleen to boost the lung; therefore, it is often used when there is spleen-lung qi deficiency, shortness of breath, palpitations, loss of appetite, loose stools, cough with empty breath, and wasting-thirst with internal heat. Besides, the *Thoroughly Revised Materia Medica* and *Orthodox Interpretation of the Materia Medica* of Qing Dynasty also clearly stated the function of Dangshen in regulating digestive symptoms and improving lung ventilation. Therefore, Ludangshen oral liquid can be used for convalescent COVID-19 patients who were of lung and spleen qi deficiency [4].

Previous laboratory studies have showed that Dangshen could adjust gastrointestinal motility, promote digestion and absorption, protect gastric mucosa, and have anti-ulcer function [12–14]. It was also found that Dangshen can improve resistance ability, and have anti-fatigue and anti-anoxia function [15, 16]. A systematic review of Dangshen indicated that Dangshen formulate can improve lung function and quality of life of patients with chronic obstructive pulmonary disease [17]. Besides, Ludangshen oral liquid can help enhance the immune activity [18, 19]. All these studies proved that Ludangshen oral liquid and its major components can help improve digestive and respiratory function and relieve related symptoms. Previous studies about recovery of COVID-19 patients more focused on description of basic characteristics and re-test RT-PCR positive risk, but very few could provide treatment information of COVID-19 during recovery period [6, 20–22].

This study with advantages used a randomized placebo-controlled design to explore the effects of Ludangshen oral liquid in convalescent COVID-19 patients, which is considered as one of the most powerful tools for interventional study [23]. During data analyses, we performed sensitivity analysis to test the robustness of the results by comparing FAS and PPS results. And there are few studies explore the effects of Chinese medicine in convalescent COVID-19 patients with symptoms related to decreased digestive and respiratory function.

The study had some limitations. The present study only included patients of Wuhan city, and therefore the conclusion may only be relevant to Chinese people. A larger sample size and longer follow-up for further verification is required.

In conclusion, the study suggested that two weeks of Ludangshen oral liquid may have certain effects on improving digestive and respiratory symptoms including fatigue, anorexia, loose stools and shortness of breath, and Ludangshen oral liquid can be one of the choices for management of convalescent COVID-19 patients.

## Conclusion

The study suggested that two weeks of Ludangshen oral liquid treatment may have certain effects for convalescent COVID-19 patients on improving digestive and respiratory symptoms including fatigue, anorexia, loose stools and shortness of breath, which may be one of the choices for management of convalescent COVID-19 patients with digestive and respiratory symptoms.

## Data Availability

Not applicable.

## Abbreviations

COVID-19: Coronavirus disease 2019
FAS: Full analysis set
PPS: Per-protocol set
TCM: Traditional Chinese medicine
RT-PCR: Reverse transcriptase-polymerase chain reaction
VAS: Visual analogue scale
SD: Standard deviation
IQR: Interquartile range
